# Developmental Trajectory of Appetitive Traits and their Bidirectional Relations with Body Mass Index from Infancy to Early Childhood

**DOI:** 10.1111/cob.12620

**Published:** 2023-09-05

**Authors:** Jenna R. Cummings, Leah M. Lipsky, Myles S. Faith, Tonja R. Nansel

**Affiliations:** 1 Social and Behavioral Sciences Branch, Division of Population Health Research, *Eunice Kennedy Shriver* National Institute of Child Health and Human Development, 6710B Rockledge Drive, Bethesda, MD, 20817, USA; 2 Department of Counseling, School, and Educational Psychology, Graduate School of Education, University at Buffalo – SUNY, 420 Bady Hall, Buffalo, NY, 14250, USA

**Keywords:** appetitive traits, bidirectional, body mass index, childhood, infancy

## Abstract

**Background:**

Appetitive traits, including food responsiveness, enjoyment of food, satiety responsiveness, and slowness in eating, are associated with childhood body mass index. Change in appetitive traits from infancy to childhood and the direction of causality between appetitive traits and body mass index are unclear.

**Objectives:**

The present study examined the developmental trajectory of appetitive traits and their bidirectional relations with body mass index, from infancy to early childhood.

**Methods:**

Mothers in the Pregnancy Eating Attributes Study and follow-up (*n* = 162) reported child appetitive traits using the Baby and Child Eating Behavior Questionnaires at ages 6 months and 3.5 years, respectively. Standardized body mass index (zBMI) was calculated from child anthropometrics. Cross-lagged panel models estimated bidirectional relations between appetitive traits and zBMI.

**Results:**

Food responsiveness, satiety responsiveness, and slowness in eating increased from infancy to early childhood. In cross-lagged panel models, lower infant satiety responsiveness (*B*±*SE* = −0.45±0.19, *p* = .02) predicted greater child zBMI. Infant zBMI did not predict child appetitive traits (*p-values* > .36).

**Conclusions:**

From infancy to early childhood, appetitive traits may amplify. Appetitive traits, particularly satiety responsiveness, appear to influence body mass index during this period, suggesting early intervention targeting these traits may reduce childhood obesity.

## Introduction

The incidence of obesity in childhood is higher, occurring at younger ages, and is more severe than two decades ago^1^. Appetitive traits, which are behavioral phenotypes including “food-approach” traits like food responsiveness and enjoyment of food, and “food-avoidant” traits like satiety responsiveness and slowness in eating, have been associated with childhood body mass index in more than 30 studies^2^. Understanding the developmental trajectory of appetitive traits, and their relations with body mass index over time, is important for identifying risk periods for childhood obesity.

Appetitive traits can be measured by the Baby Eating Behavior Questionnaire in infancy^3^, the Child Eating Behavior Questionnaire in childhood^4^, and the Adult Eating Behavior Questionnaire in adolescence and adulthood^5^. In infants and children, slowness in eating has also been measured by observational methods in the laboratory^6,7^. Previous studies have examined the developmental trajectory of appetitive traits measured by the Child Eating Behavior Questionnaire during middle childhood. In children studied from ages 4 to 11 years, there were small- to medium-sized positive correlations within their appetitive traits over time, increases in their food-approach traits, and decreases in their food-avoidant traits, though findings on change in their enjoyment of food were mixed^8–10^. Studies on the developmental trajectory of appetitive traits during other sensitive periods are needed. 

How children eat may change from infancy to childhood because children are rapidly developing motor and verbal skills and, for the first time, children are experiencing psychophysiological effects of solid food and are learning how to eat solid food from caregivers^11^. However, few studies have examined the developmental trajectory of appetitive traits during this period. Greater infant appetite at ages 6 weeks and 12 months measured with a single item (“At present, how is your baby/child’s appetite?”) was modestly correlated with greater enjoyment of food, quicker eating rates, and lower satiety responsiveness measured by the Child Eating Behavior Questionnaire at ages 5-6 years^12^. Except for one study finding moderate stability of appetitive traits from ages 3 months to 3 years in a sample of low-income Hispanic children^13^, correlation and change in appetitive traits measured by the Baby and Child Eating Behavior Questionnaires from infancy to childhood remain unexamined.

Relations of appetitive traits with body mass index in children may vary by age. Associations of appetitive traits at age 3 months with body mass index at age 9 months were stronger than associations of infant body mass index with later appetitive traits^14^, suggesting that appetitive traits influence body mass index with a weaker reciprocal effect. However, bidirectional associations between appetitive traits and body mass index in children were observed from ages 6 to 8 years,^15^ and associations of appetitive traits at ages 4 and 6 years with body mass index at ages 10 and 14 years were mostly non-significant^16,17^. Although these findings are from observational research, taken together, they suggest a causal influence of appetitive traits on body mass index that would be stronger earlier in development. Studies following infants through childhood have not examined possible reciprocal effects between appetitive traits and body mass index^13,18^.

The present study examined the developmental trajectory of appetitive traits, and their bidirectional relations with body mass index, from infancy (age 6 months) to early childhood (age 3.5 years). Based on findings in older childhood, we hypothesized positive correlations within appetitive traits over time and we hypothesized food-approach traits would increase and food-avoidant traits would decrease between infancy and early childhood. Given that the influence of appetitive traits on body mass index may be stronger earlier in development, we hypothesized the prospective association of infant appetitive traits with early child body mass index would be stronger than the prospective association of infant body mass index with early child appetitive traits.

## Methods

### Participants and Procedure

Data are from the Pregnancy Eating Attributes Study (PEAS) and the Sprouts follow-up study. PEAS investigated reward-related eating, diet quality, and weight during pregnancy and postpartum in women receiving prenatal care in Chapel Hill, North Carolina, United States^19^. For full sociodemographic information regarding the PEAS sample, please see Nansel et al., 2020^20^. Sprouts is an ongoing study investigating eating behaviors, diet quality, and weight in the offspring from this cohort, beginning when children are age 3.5 years and continuing annually through age 7 years. The present study was a secondary data analysis using available data on appetitive traits and body mass index from PEAS and Sprouts.

Pregnant women were identified through the electronic medical records database. Exclusion criteria for PEAS included BMI < 18.5 kg/m^2^, pre-existing diabetes, any medical condition contraindicating study participation, participant-reported history of eating disorder, and medication use that could affect diet or weight. Mothers in PEAS who provided consent to be contacted for future studies were re-recruited for Sprouts; additional exclusion criteria for Sprouts were child neurocognitive disability or attention deficit/hyperactivity disorder. A flow chart illustrating the number of participants at each stage of PEAS was published^20^. A total of 321 mothers completed PEAS through one-year postpartum and 162 mother-child dyads enrolled in Sprouts when children were age 3.5 years.

The University of North Carolina Institutional Review Board approved procedures (study #18-2030) in accordance with the ethical standards of the Helsinki Declaration of 1975 as revised in 1983. Mothers provided informed consent for herself and her child. Sprouts procedures included in-person assessments; however, for the initial Sprouts assessment, 19% of participants completed remotely due to the COVID-19 shutdown.

### Measures

#### Appetitive Traits

Mothers reported on their child’s appetitive traits at age 6 months via the 18-item Baby Eating Behavior Questionnaire^3^ and at age 3.5 years via the 35-item Child Eating Behavior Questionnaire^4^. The Baby Eating Behavior Questionnaire measures food responsiveness (e.g., “If given the chance, my baby would always be feeding”; 6 items; *α* = 0.80), enjoyment of food (e.g., “My baby loved milk”; 4 items; *α* = 0.75), satiety responsiveness (e.g., “My baby got full before taking all the milk I thought s/he should have”; 3 items; *α* = 0.39), and slowness in eating (e.g., “My baby fed slowly”; 4 items; *α* = 0.62) in infants when they are milk feeding. The Child Eating Behavior Questionnaire measures food responsiveness (e.g., “If given the chance, my child would always have food in his/her mouth”; 5 items; *α* = .61), enjoyment of food (e.g., “My child enjoys eating”; 4 items; *α* = .65), satiety responsiveness (e.g., “My child gets full before his/her meal is finished”; 5 items; *α* = .69), and slowness in eating (e.g., “My child eats more and more slowly during the course of a meal”; 4 items; *α* = .68) in children. The appetitive traits of desire to drink, emotional over-eating, emotional under-eating, and food fussiness were not investigated in the present study because they are assessed by the Child Eating Behavior Questionnaire, but not the Baby Eating Questionnaire, so their developmental trajectory and bidirectional relations with body mass index could not be examined. For both questionnaires, mothers rated items on a 5-point Likert scale (1 = “Never,” 5 = “Always”). Items were reverse scored where appropriate and items for each appetitive trait were averaged at ages 6 months and 3.5 years. Higher scores indicated greater food responsiveness, enjoyment of food, and satiety responsiveness and slower eating rates.

#### Anthropometrics

At age 6 months, weight was measured on an infant scale (Tanita BD-585) and length was measured using a recumbent infant board with a stadiometer (Pediatric Lengthboard-Ellard Instrumentation PED LB 35-107-X). At age 3.5 years, weight was measured using a calibrated scale (SECA 813) and height was measured using a stadiometer (SECA 213). All weight and length/height measurements were to the nearest 0.01 kg and 0.1 cm, respectively. Anthropometrics were collected in duplicate; a third measure was obtained if the first two measures were discrepant by > 0.2 kg or 1 cm in infancy and by > 1 kg or 1 cm in early childhood. The two closest measurements were averaged. Mothers provided height and weight measurements for children who participated remotely (*n* = 24); of these, 9 indicated the height and weight measurements were from recent medical visits.

Weight-for-length z-scores (WFLz) and z-scored Body Mass Index (zBMI) were calculated based on the US Centers for Disease Control and Prevention reference growth curves for age and sex assigned at birth^21^. Although the World Health Organization recommends using WFLz for age 0 to 2 years, and zBMI for > age 2 years, findings from multiple studies support the validity of using zBMI for children < age 2 years^22–25^. Consistent with prior research^22^, correlations between WFLz and zBMI in this sample were > .99, so zBMI was used to assess longitudinal patterns in body mass index.

#### Potential Confounders

Given that household income and breastfeeding duration may affect both appetitive traits^26,27^ and body mass index^28,29^, income-poverty ratio and exclusive breastfeeding duration were considered as confounders. Mothers reported total annual household income in the past year, including income earned by those >18 years of age in the household and not including assets, and reported total people and children (<18 years of age) living in the household. Income-poverty ratio was calculated by dividing maternal-reported total annual household income at baseline by the US Census Bureau 2016 poverty thresholds, accounting for household size and number of children^30^. During postpartum, mothers reported when they introduced different feeding modes to their infant^31^. Exclusive breastfeeding duration was calculated by determining the number of months mothers breastfed their infant or fed their infant breastmilk from a bottle, with no formula or complementary food feeding.

### Statistical Analysis

Statistical analysis was completed in R 4.2.1 (Vienna, Austria). Statistical significance was set at *p* < .05. Paired sample *t*-tests were conducted to assess mean-level change in appetitive traits and zBMI from infancy to early childhood. To inform which potential confounders were retained as covariates in subsequent models, bivariate correlations (uncorrected for multiple comparisons) were conducted. Cross-lagged panel modeling was conducted using the lavaan package. Full information maximum likelihood (FIML) estimation, which allows retention of the maximum number of valid cases while producing unbiased estimates, was applied to account for missing data. Model fit was considered acceptable based on the following established criteria^32^: comparative fit index (CFI) > .95 and root mean square error of approximation (RMSEA) < .06. The cross-lagged panel model simultaneously estimated (1) the cross-sectional association of appetitive traits and zBMI in infancy, (2) the prospective association of infant appetitive traits with child appetitive traits, (3) the prospective association of infant zBMI with child zBMI, (4) the cross-lagged prospective association of infant appetitive traits with child zBMI, (5) the cross-lagged prospective association of infant zBMI with child appetitive traits, and (6) the cross-sectional associations of appetitive traits and zBMI in early childhood. Separate models were estimated for each appetitive trait.

## Results

### Participants and Sociodemographic Variables

Table 1 provides child sex assigned at birth, child age in months, and means and standard deviations for variables of interest at each assessment. Mothers were on average age 30.46±2.65 years at baseline and maternal race breakdown was 67.3% non-Hispanic White and 32.7% minority race, including Black, Asian, Hispanic, and/or Latino. Average household income-poverty ratio at baseline was 3.84±1.97, indicating $77,414.40 per year for a family of 3 and $93,312 per year for a family of 4. Children were exclusively breastfed for an average of 2.00±2.65 months. Although household income-poverty ratio was significantly correlated with infant slowness in eating (*r* = .13, *p* = .048), child desire to drink (*r* = −.36, *p* < .001), and child satiety responsiveness (*r* = .19, *p* = .021), and exclusive breastfeeding duration was significantly correlated with infant food responsiveness (*r* = −.20, *p* = .003) and child desire to drink (*r* = −.20, *p* = .013), these variables were not correlated with infant or child zBMI (*p-value*s > .36), so they were not included as covariates in subsequent models.

### Mean-Level Change in Appetitive Traits from Infancy to Early Childhood

Table 2 presents estimates from paired sample *t*-tests. All appetitive traits showed significant mean-level change over time. There was a small increase in food responsiveness, a large decrease in enjoyment of food, a large increase in satiety responsiveness, and a medium increase in slowness in eating from infancy to early childhood. There also was a small increase in zBMI from infancy to early childhood.

### Cross-Lagged Panel Models

**Figures 1:**
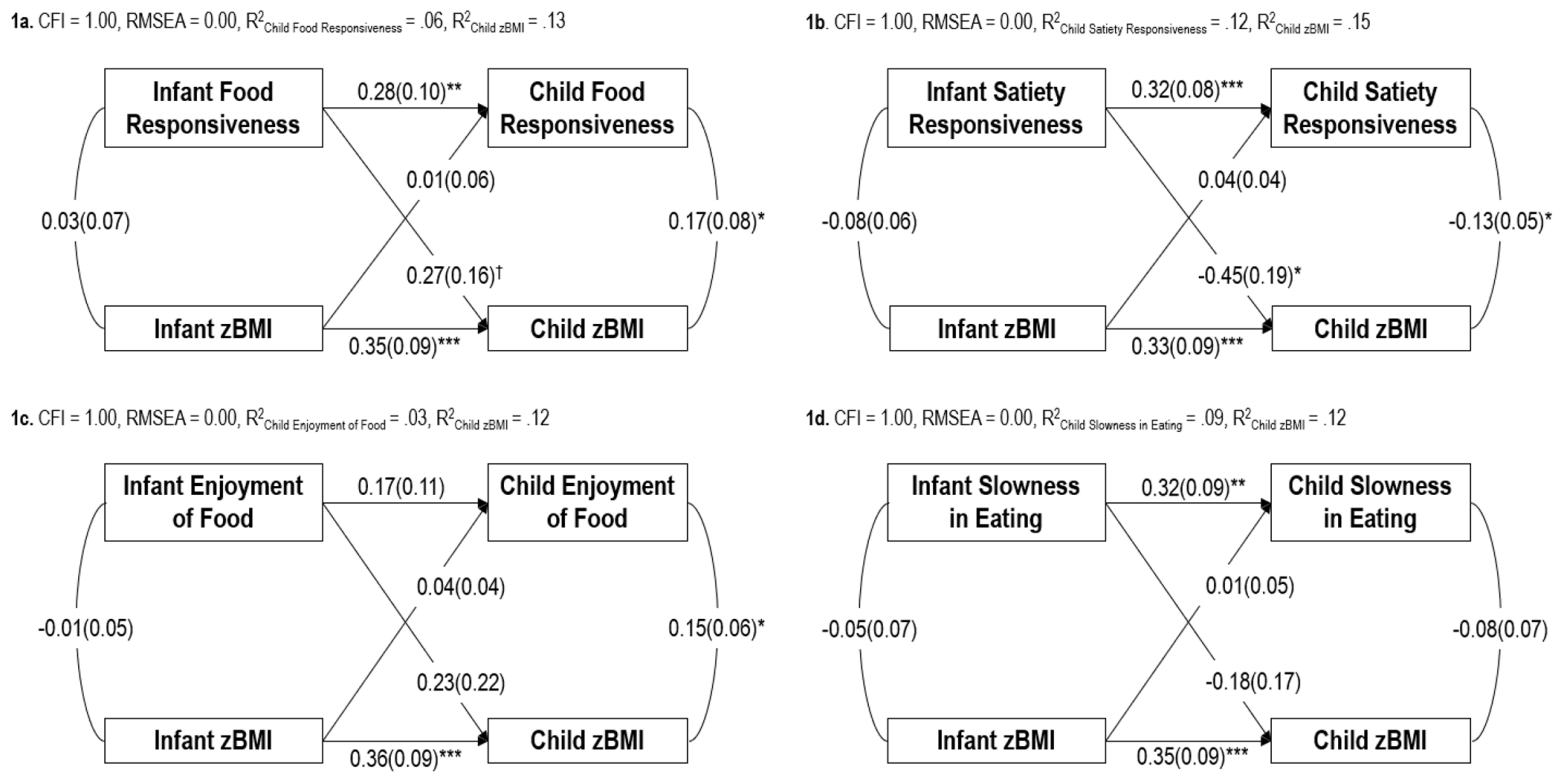
a-d: Cross-lagged panel models of relations between appetitive traits and zBMI from infancy to early childhood. CFI = Comparative Fit Index, RMSEA = Root Mean Square Error of Approximation, ***p < .001, **p < .01, *p < .05, ^†^p < .10

Figures 1a–d present estimates from the cross-lagged panel models assessing relations between appetitive traits and zBMI over time. Model fits were good, and models accounted for 3-12% of the variance in appetitive traits at age 3.5 years and 12-15% of the variance in body mass index at age 3.5 years. There were no statistically significant cross-sectional associations of appetitive traits and zBMI at age 6 months.

Greater infant food responsiveness, satiety responsiveness, and slowness in eating at age 6 months significantly predicted greater child food responsiveness, satiety responsiveness, and slowness in eating at age 3.5 years, respectively. The prospective association of infant enjoyment of food at age 6 months with child enjoyment of food at age 3.5 years was non-significant. Greater infant zBMI at age 6 months significantly predicted greater child zBMI at age 3.5 years.

In the cross-lagged prospective associations, lower infant satiety responsiveness at age 6 months significantly predicted greater child zBMI at age 3.5 years. The associations of infant food responsiveness, enjoyment of food, and slowness in eating at age 6 months with child zBMI at age 3.5 years were non-significant, though the association of greater infant food responsiveness at age 6 months with greater child zBMI at age 3.5 years approached statistical significance (*p* = .09). Infant zBMI at age 6 months did not significantly predict any child appetitive trait at age 3.5 years.

Greater child food responsiveness and enjoyment of food were significantly cross-sectionally associated with greater child zBMI at age 3.5 years, and greater child satiety responsiveness was significantly cross-sectionally associated with lower child zBMI at age 3.5 years. The cross-sectional association of slowness in eating and zBMI at age 3.5 years was non-significant.

Estimates from the cross-lagged panel models assessing relations between appetitive traits and zBMI over time including only children with measured height and weight were similar to those in the full sample (online supporting information).

## Discussion

Satiety responsiveness, slowness in eating, and food responsiveness were positively correlated within trait over time and increased from age 6 months to 3.5 years in 162 children living in North Carolina, United States. Enjoyment of food decreased during this period and was not significantly correlated within trait over time. While lower infant satiety responsiveness predicted greater child zBMI, infant zBMI did not significantly predict child satiety responsiveness. Prospective associations of greater infant food responsiveness, greater infant enjoyment of food, and lower infant slowness in eating with greater zBMI were larger in magnitude than the reciprocal associations, though they did not reach statistical significance. Overall, infancy to early childhood may be a sensitive developmental period when most appetitive traits increase and when satiety responsiveness protects against childhood obesity.

These findings advance understanding of the developmental trajectory of appetitive traits from infancy to early childhood. Consistent with a prior study in low-income Hispanic children^13^, positive correlations within food responsiveness, satiety responsiveness, and slowness in eating from infancy to early childhood provide evidence of reliability in measurement over time and across the Baby and Child Eating Behavior Questionnaires. The stability of appetitive traits likely reflects the substantial genetic influence on appetitive traits^33,34^; however, the small magnitude of correlations within appetitive traits indicates intra-individual variation over time, which may reflect changes in environmental influences such as feeding mode during infancy^26,27,35^ and parental feeding styles and practices during early childhood^36^. 

In contrast to trajectories previously shown from age 4 to 11 years—whereby food-avoidant traits decreased and food-approach traits increased^8–10^—satiety responsiveness and slowness in eating (food-avoidant traits) increased from infancy to early childhood to a greater extent than food responsiveness (a food-approach trait), which increased modestly. These findings may suggest that development of food-avoidant traits from infancy to early childhood is distinct from that in older childhood. It is possible that as children begin eating solid foods the behavioral expressions of satiety responsiveness (e.g., child declines meal after snack) and slowness in eating (e.g., child finishes a meal slowly) amplify along with food responsiveness (e.g., child asks for more food). Alternatively, observed changes could reflect other aspects of aging; for example, children speaking more. Given that appetitive traits were measured by maternal report, observed changes may also reflect changes in mothers’ perceptions of her child’s eating behavior across this developmental period.

Enjoyment of food was not significantly correlated from infancy to early childhood and was the only appetitive trait that decreased in magnitude, suggesting that the expression of enjoyment of food differs substantively from infancy to early childhood or that the Baby and Child Eating Behavior Questionnaires assess different forms of enjoyment of food. Prior studies have similarly indicated low stability of enjoyment of food from infancy to early childhood^13^ and mixed findings on change in enjoyment of food in older childhood^8–10^. Future investigations of correlations within appetitive traits measured by the Baby, Child, and Adult Eating Behavior Questionnaires would strengthen the literature and determine whether these different questionnaires are assessing the same constructs throughout different developmental stages.

The present study found that greater infant satiety responsiveness predicted lower child zBMI, with no reciprocal influence, supporting a core hypothesis of the behavioral susceptibility theory of obesity: that appetitive traits influence susceptibility to weight gain in an obesogenic environment. Other studies have found bidirectional associations between appetitive traits and body mass index^15^ or that body mass index influences appetitive traits^16,17^, but these studies investigated relations during older childhood (ages 4 to 14 years). Indeed, the present study findings are consistent with a prior study showing that appetitive traits had a stronger influence on body mass index versus the reciprocal from ages 3 to 15 months^14^. Future longitudinal research examining bidirectional relations between appetitive traits and body mass index from infancy to adulthood could verify whether a causal role of appetitive traits in weight gain is limited to early in development.

Findings should be interpreted in light of strengths and limitations of the study. Internal validity was strengthened by the prospective study design and the cross-lagged panel modeling approach with modern statistical approaches that reduce bias from data missingness. Although COVID shutdowns prevented investigators from directly measuring a subsample of children’s height and weight, findings in the analysis constrained to children with measured height and weight were consistent with those from the full sample. Given that the Baby and Child Eating Behavior Questionnaires are parent-report, measurement of appetitive traits may be subject to bias introduced by mothers’ interpretation of their child’s appetite. The generalizability of the results is limited by the representation of individuals in the geographic area of recruitment and loss to follow-up.

In conclusion, food-approach and food-avoidant traits may increase from infancy to early childhood and influence body mass index early in development. Future longitudinal research investigating the developmental trajectory of appetitive traits from infancy through adulthood would further inform risk and protective periods for obesity across the lifespan. Determining methods to increase satiety responsiveness from infancy to early childhood may be critical for preventing childhood obesity. Prior research suggests maternal pregnancy diet^37^ as well as breastfeeding versus formula feeding^38^ may influence satiety responsiveness in infants. Targeting infants with lower satiety responsiveness in childhood obesity prevention efforts may also be beneficial.

## Supplementary Material

Supinfo

## Figures and Tables

**Table 1:** Child Sex Assigned at Birth, Age in Months, and Descriptives for Variables of Interest.

	Age 6 Months		Age 3.5 Years	

	*n*	*%* or *M(SD)*	*n*	*%* or *M(SD)*
**Child Sex Assigned at Birth**				
Female	147	50.7%	83	51.6%
Male	143	49.3%	79	48.4%
**Child Age (in months)**	290	6.29 (0.57)	161	42.18 (2.23)
**Child Appetitive Traits**				
Food Responsiveness	229	2.23 (0.65)	160	2.48 (0.73)
Enjoyment of Food	229	4.49 (0.47)	159	3.80 (0.61)
Satiety Responsiveness	229	2.24 (0.59)	160	3.07 (0.53)
Slowness in Eating	229	2.40 (0.62)	160	3.08 (0.67)
**Child Body Mass index**				
zBMI	290	−0.16 (1.14)	150	0.16 (1.24)

zBMI = Standardized Body Mass Index

**Table 2:** Mean-Level Change in Appetitive Traits from Infancy to Early Childhood.

	Paired Differences			

	*M±SE*	*t*	*p*	Cohen’s *d*
**Child Appetitive Traits**				
Food Responsiveness	0.26±0.07	3.47	<.001	0.30
Enjoyment of Food	−0.68±0.06	−11.19	<.001	−0.98
Satiety Responsiveness	0.90±0.05	16.89	<.001	1.47
Slowness in Eating	0.61±0.07	9.03	<.001	0.79
**Child Body Mass Index**				
zBMI	0.32±0.12	2.78	.006	0.23

zBMI = Standardized Body Mass Index
