# Supplementary material for: Developmental Trajectory of Appetitive Traits and their Bidirectional Relations with Body Mass Index from Infancy to Early Childhood
Source: Clin Obes. Author manuscript; Available in PMC 2025 Feb 1. (PMC10841422; doi:10.1111/cob.12620)

Manuscript Title: Developmental Trajectory of Appetitive Traits and their Bidirectional Relations with Body Mass Index from Infancy to Early Childhood

Authors: Jenna R. Cummings[1a], Leah M. Lipsky[1], Myles S. Faith[2], & Tonja R. Nansel[1]

[1]Social and Behavioral Sciences Branch, Division of Population Health Research, *Eunice Kennedy Shriver* National Institute of Child Health and Human Development, 6710B Rockledge Drive, Bethesda, MD, 20817, USA

[2]Department of Counseling, School, and Educational Psychology, Graduate School of Education, University at Buffalo – SUNY, 420 Bady Hall, Buffalo, NY, 14250, USA

[a]Jenna R. Cummings is now at the Department of Psychology, University of Liverpool, 74 Bedford St. S, Liverpool, L69 7ZQ, UK

Corresponding author: Tonja R. Nansel. Mailing address: 6710B Rockledge Drive, Bethesda, MD, 20817, USA. Telephone number: (301) 435-6937. E-mail address: nanselt@mail.nih.gov.

Online Supporting Information

Figures S1a-d present estimates from the cross-lagged panel models assessing relations between appetitive traits and zBMI over time, only including children with measured height and weight. Model fits were good, and models accounted for 2-14% of the variance in appetitive traits at age 3.5 years and 16-28% of the variance in body mass index at age 3.5 years. There were no statistically significant cross-sectional associations of appetitive traits and zBMI at age 6 months.

Greater infant food responsiveness, satiety responsiveness, and slowness in eating at age 6 months significantly predicted greater child food responsiveness, satiety responsiveness, and slowness in eating at age 3.5 years, respectively. The prospective association of infant enjoyment of food at age 6 months with child enjoyment of food at age 3.5 years was non-significant. Greater infant zBMI at age 6 months significantly predicted greater child zBMI at age 3.5 years.

In the cross-lagged prospective associations, greater infant enjoyment of food and lower infant satiety responsiveness at age 6 months significantly predicted greater child zBMI at age 3.5 years. The associations of infant food responsiveness and slowness in eating at age 6 months with child zBMI at age 3.5 years were non-significant, though the association of greater infant food responsiveness at age 6 months with greater child zBMI at age 3.5 years approached statistical

significance. Infant zBMI at age 6 months did not significantly predict any child appetitive trait at age 3.5 years.

Greater child satiety responsiveness and slowness in eating were significantly cross-sectionally associated with lower child zBMI at age 3.5 years. The cross-sectional associations of food responsiveness and enjoyment of food with zBMI at age 3.5 years were non-significant, though the association of greater child enjoyment of food with greater child zBMI at age 3.5 years approached statistical significance.

Online Supporting Information Figures S1a-d. Cross-lagged panel models of relations between appetitive traits and zBMI from infancy to early childhood only including children with measured height and weight. CFI = Comparative Fit Index, RMSEA = Root Mean Square Error of Approximation, ***p < .001, **p < .01, *p < .05, †p < .10

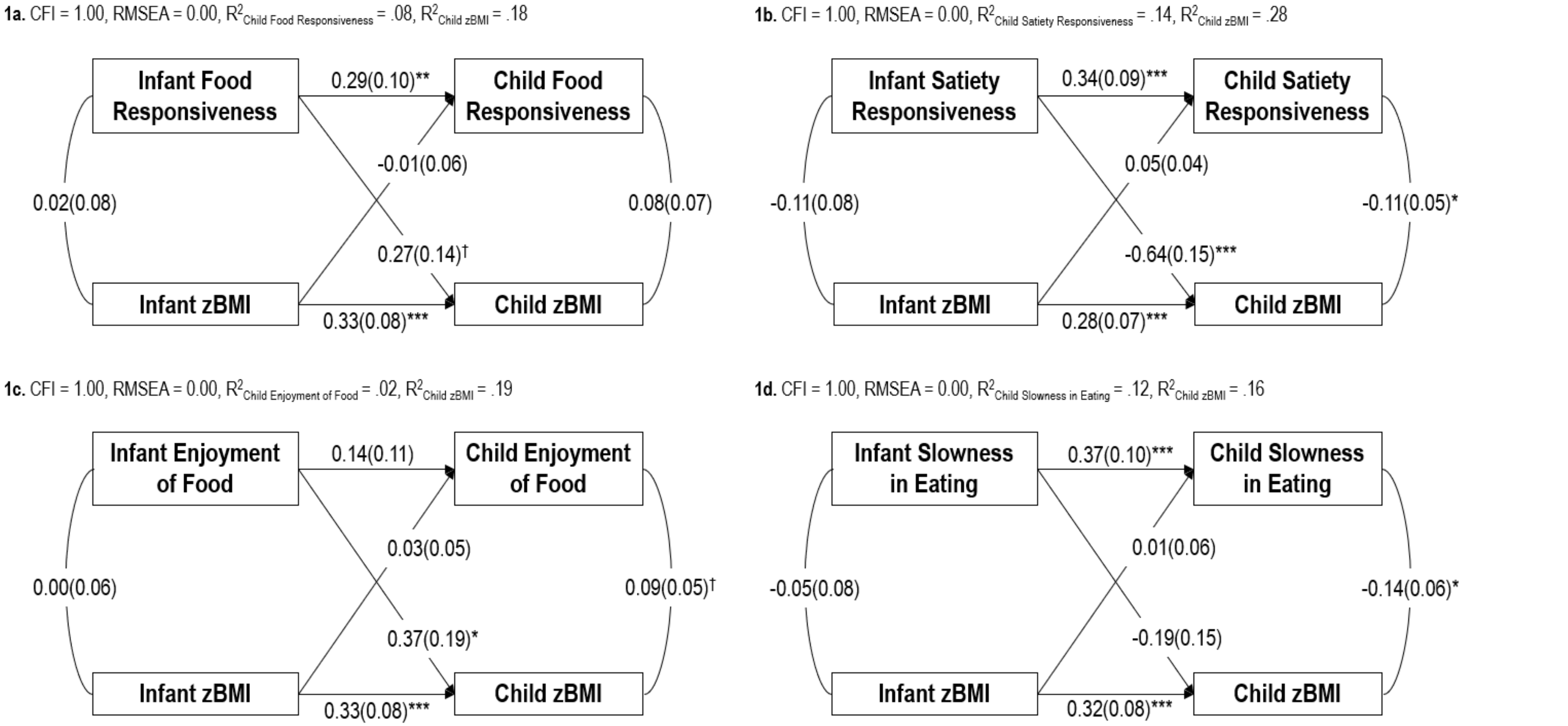

Supplement: Supinfo [file NIHMS1939184-supplement-Supinfo.pdf]
